# Inoculation Gone Mad

**Published:** 1886-03

**Authors:** 


### INOCULATION GONE MAD--WHAT IT WILL COME TO.

Oh, the doctors now a look of wisdom wear,
As with earnestness together they declare,
That the only sure salvation
Is in quick inoculation,
When a deadly scourge develops anywhere.

They'll inoculate for cholera, and when
There's diphtheria about they'll cut again ;
And, in saving you from harm,
They will carve your upper arm
Till you think you have escaped a lion's den.

Against hydrophobia and fever too,
They will wisely undertake to poison you,
And, it seemeth very clear,
In the future we shall hear,
Of this remedy for pure mania a potu.

They will give it for sweet love's consuming fire,
For red-countenanced and eyeball-glaring ire,
And the evil of divorce
They will cure by this, of course,
And prevent the fabrications of the liar.

—*Columbus Dispatch.*

The U. S. Government should send "a commission" to investigate the above "methods"—and by all means get Holt of it, if it will "take"—especially that method of inoculating that will "prevent the fabrications."

